# Pre-Visual Spectral Responses of Pine Trees Affected by Pine Wilt Disease Revealed by Onset-Aligned UAV Multispectral Monitoring

**DOI:** 10.3390/plants15172720

**Published:** 2026-09-04

**Authors:** Ziyi You, Nan Zheng, Zuliang Jiang, Runkai Chen, Zhixuan Ye, Songqing Wu, Run Yu, Feiping Zhang

**Affiliations:** State Key Laboratory of Agricultural and Forestry Biosecurity, College of Forestry, Fujian Agriculture and Forestry University, Fuzhou 350002, China

**Keywords:** pine wilt disease, *Pinus massoniana*, UAV multispectral remote sensing, pre-visual detection, vegetation indices, onset-aligned analysis, forest disease monitoring

## Abstract

Early detection of pine wilt disease (PWD) before visible crown discoloration is important for field inspection, confirmatory sampling, and timely management. However, because individual trees reach visible discoloration at different times, calendar-based analyses can blur the development of pre-visual spectral responses. Weekly UAV multispectral data from two *Pinus massoniana* forest sites were therefore aligned to the first visible crown discoloration of each tree. Crown-level band reflectance and vegetation indices (VIs) were examined from eight to one weeks before first visible discoloration, and healthy reference observations at each relative week were used to define 5th–95th percentile reference ranges for detection-rate (DR) analysis. Several VIs showed significant group-level differences from approximately seven weeks before first visible discoloration, whereas mean VI-based DR increased markedly to 0.52 at two weeks and 0.68 at one week before discoloration; single-band DRs were less stable. These results indicate a progressive pre-visual spectral response, with an exploratory early group-level indication emerging approximately seven weeks before discoloration and a stronger, more consistent spectral departure during the final two weeks. This onset-aligned framework provides a practical basis for scheduling repeated UAV surveillance and prioritizing field verification before obvious symptoms emerge.

## 1. Introduction

Pine wilt disease (PWD), caused by the pine wood nematode *Bursaphelenchus xylophilus*, is one of the most destructive diseases threatening pine forest ecosystems, owing to its rapid spread and high tree mortality [1,2]. Once affected trees exhibit visible crown discoloration, wilting, or other external symptoms, disease development has usually reached an advanced stage, leaving limited time for field inspection, confirmatory sampling, and management intervention [3]. Detecting spectral changes before obvious crown symptoms appear may therefore provide additional time for identifying high-risk trees and organizing subsequent field verification [4].

Remote sensing provides an efficient means of monitoring forest pests and diseases over large and spatially heterogeneous areas [5,6]. Unmanned aerial vehicle (UAV) imagery is particularly suitable for individual-tree and crown-level monitoring because of its high spatial resolution, operational flexibility, and short revisit interval [7,8]. Multispectral sensors record visible, red-edge, and near-infrared reflectance, from which vegetation indices (VIs) associated with canopy greenness, pigment status, and vegetation vitality can be derived [9,10]. Changes in these spectral features may occur before obvious crown discoloration and thus provide non-destructive indicators of developing tree stress. Previous studies have demonstrated the potential of UAV multispectral and hyperspectral imagery for distinguishing healthy and PWD-affected trees and for detecting spectral changes before visible symptoms [11,12,13,14].

Previous studies have identified favorable calendar periods, disease stages, or spectral features for the early detection of PWD using single-date or multi-temporal UAV imagery [15,16,17,18]. However, these monitoring windows have generally been defined according to acquisition date or broad disease-stage categories. Individual trees may differ in infection timing, disease progression rate, and the onset of visible crown discoloration. Consequently, trees observed on the same calendar date may occupy different positions along the disease-development trajectory, while trees at a similar pre-visual stage may be distributed across different acquisition dates. Calendar-based grouping may therefore obscure the temporal relationship between spectral deviation and the first visible crown change. It remains necessary to determine how early spectral differences emerge relative to tree-specific visible discoloration and when these differences become sufficiently widespread to produce a marked increase in the proportion of spectrally abnormal trees.

A critical unresolved issue in field surveillance of PWD is not simply whether affected trees can be distinguished from healthy trees, but how much time UAV monitoring can provide before obvious crown discoloration develops. This distinction is important because the earliest statistically detectable group-level shift may occur well before spectral abnormalities become sufficiently widespread among individual trees to support targeted field inspection. To address this management-relevant temporal question, we reorganized weekly UAV multispectral observations from two *Pinus massoniana* forest sites using the first visible crown discoloration of each subsequently discolored tree as an individual temporal anchor. Crown-level band reflectance and VIs were retrospectively aligned from eight to one weeks before first visible discoloration and compared with week-specific healthy reference observations. We specifically examined (1) how early group-level spectral differences emerged and (2) when the proportion of subsequently discolored trees outside the healthy spectral range increased markedly. By separating the initial emergence of spectral deviation from its later strengthening, this study provides a disease-progress-relative framework for defining the pre-visual warning window of PWD and for improving the timing of repeated UAV surveillance, field inspection, and confirmatory sampling.

## 2. Results

### 2.1. Temporal Patterns of Band Reflectance Before First Visible Discoloration

**Figure 1:**
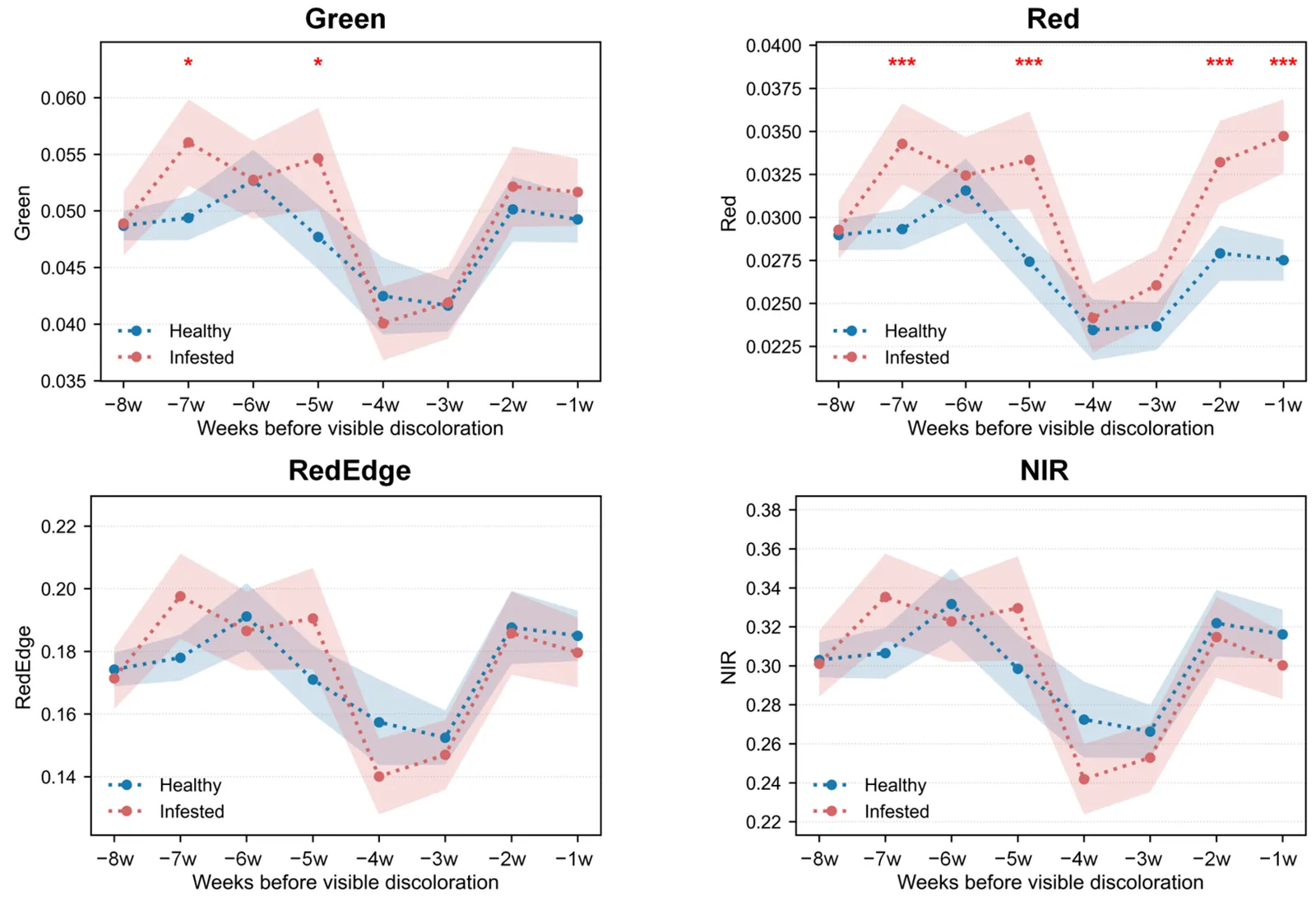
Time-series changes in band reflectance before visible discoloration. Points and shaded areas represent group means and 95% confidence intervals, respectively. Asterisks indicate BH–FDR-adjusted significance levels from two-sided Mann–Whitney U tests: *, adjusted *p* < 0.05; and ***, adjusted *p* < 0.001.

Band-reflectance differences between healthy and PWD-consistent observations varied among spectral bands and relative times (Figure 1). Green reflectance was significantly higher in PWD-consistent samples at seven and five weeks before first visible crown discoloration. Red reflectance showed more frequent group differences and was significantly higher in PWD-consistent samples at seven, five, two, and one weeks before first visible discoloration. In contrast, no consistent significant differences were observed for the red-edge or NIR bands.

The trajectories of all four bands fluctuated across the pre-visual period rather than showing a continuous increase in group separation as first visible discoloration approached. Among the individual bands, red reflectance showed the most recurrent differences between the two sample groups, whereas the temporal responses of green, red-edge, and NIR reflectance were less consistent.

### 2.2. Selection of Representative VIs

**Figure 2:**
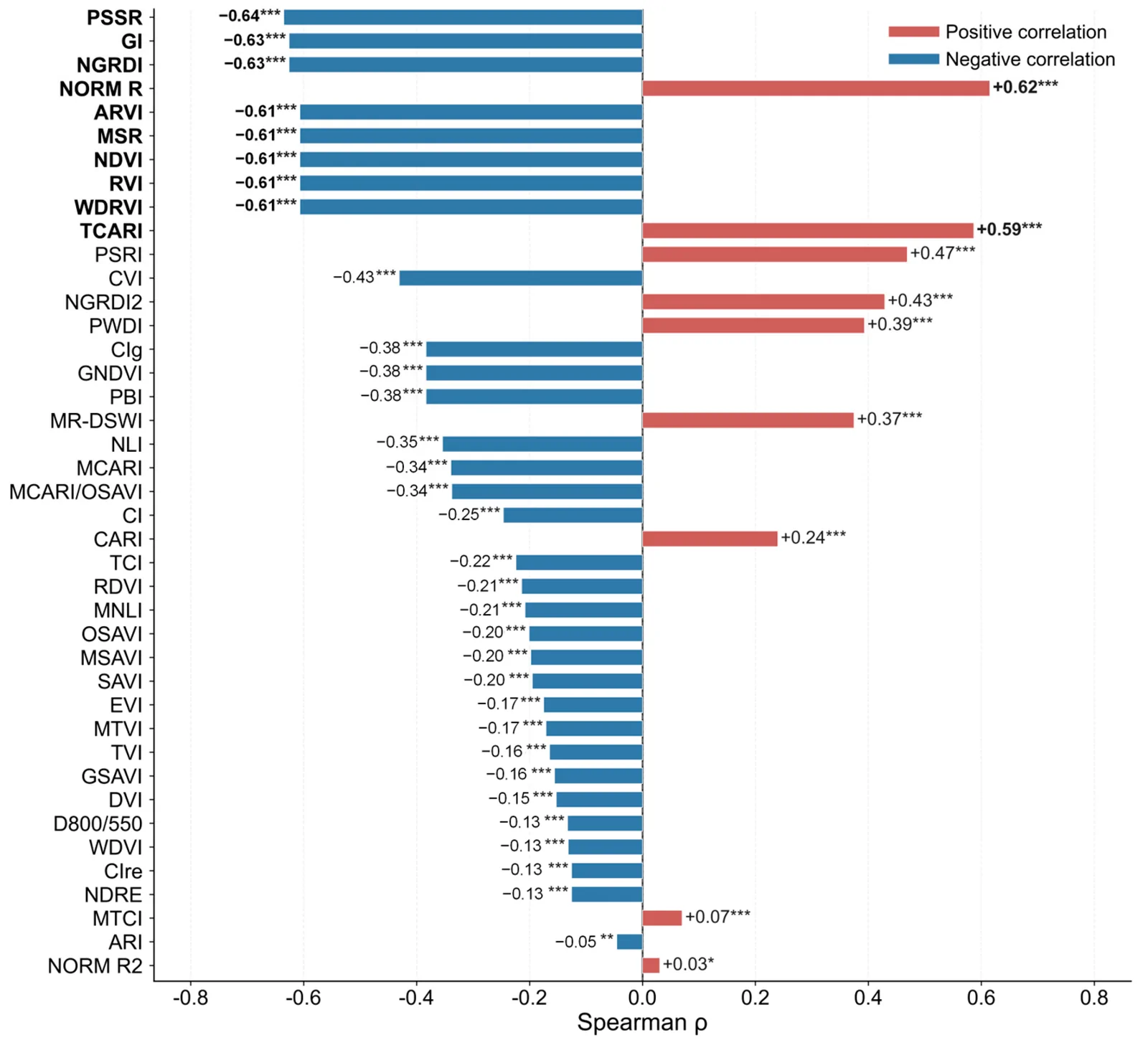
Spearman correlation between candidate VIs and sample status. *, adjusted *p* < 0.05; **, adjusted *p* < 0.01; ***, adjusted *p* < 0.001.

The direction and magnitude of the Spearman correlations between candidate VIs and sample status varied considerably (Figure 2). PSSR, GI, NGRDI, ARVI, MSR, NDVI, RVI, and WDRVI were negatively correlated with sample status, with absolute correlation coefficients exceeding 0.61. In contrast, NORM R and TCARI were positively correlated with sample status, with correlation coefficients of 0.62 and 0.59, respectively. All ten correlations remained significant after BH–FDR correction. Because these indices had the ten largest absolute correlation coefficients among the candidate VIs, they were retained for subsequent temporal, significance, and DR analyses. The stability of this exploratory feature-ranking procedure was further evaluated using 100 tree-level bootstrap resamples (Table 1). Across the 100 bootstrap runs, the ten selected VIs were retained in the Top 10 in 98–100% of runs, indicating high stability of the selected feature set under tree-level resampling. Nevertheless, this resampling analysis was used to assess ranking stability rather than to provide independent validation of predictive performance.

### 2.3. Temporal Responses of Representative VIs

**Figure 3:**
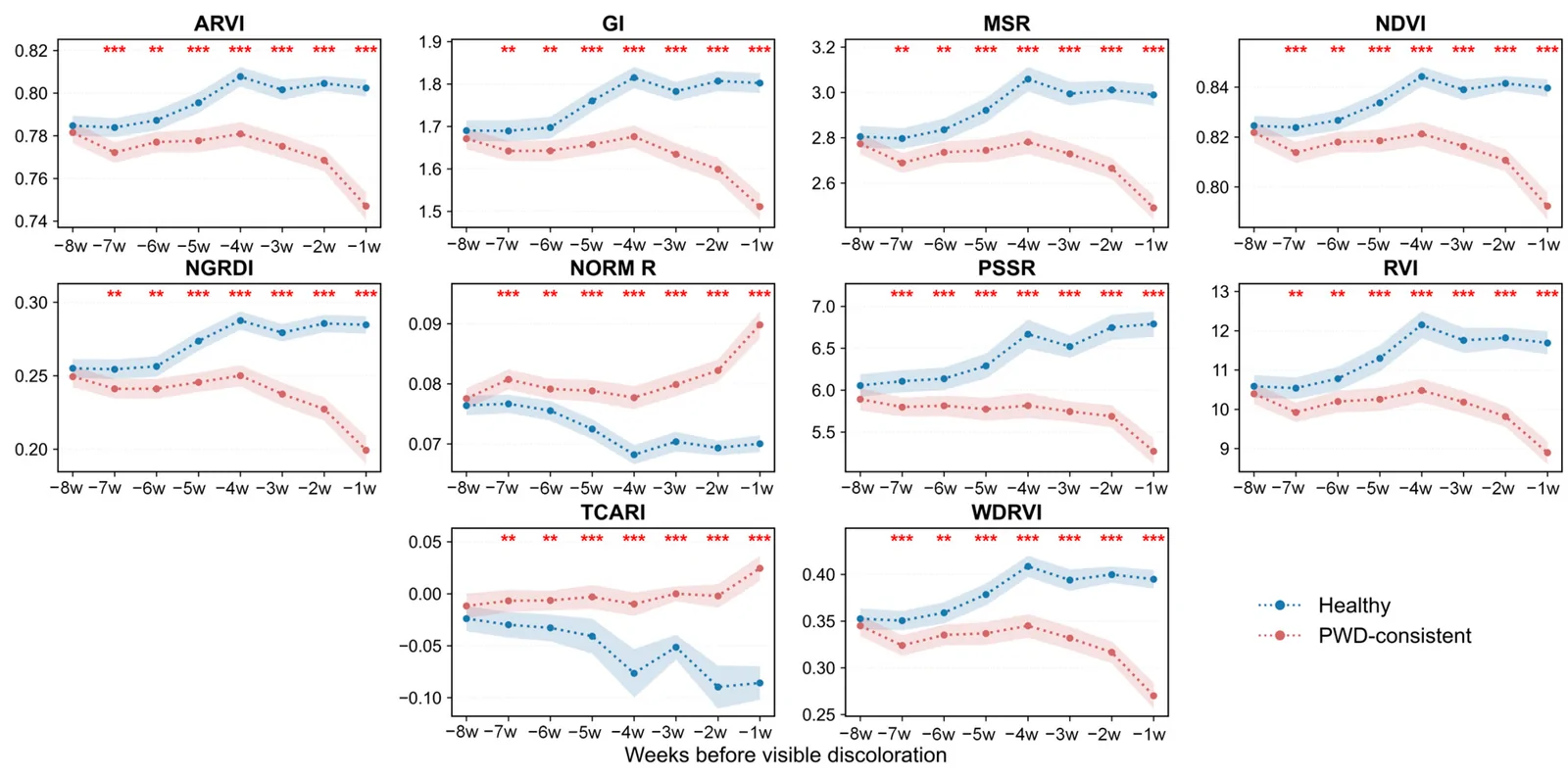
Time-series changes in representative VIs before visible discoloration. Points and shaded areas represent group means and 95% confidence intervals, respectively. Asterisks indicate BH–FDR-adjusted significance levels from two-sided Mann–Whitney U tests: **, adjusted *p* < 0.01; and ***, adjusted *p* < 0.001.

The selected VIs showed more temporally consistent differences between healthy and PWD-consistent observations than the individual bands (Figure 3). For most VIs, significant group differences first appeared approximately seven weeks before first visible crown discoloration and were observed at most subsequent relative times.

ARVI, GI, MSR, NDVI, NGRDI, PSSR, RVI, and WDRVI were generally lower in PWD-consistent observations than in healthy samples. The differences between the two groups became more pronounced as first visible discoloration approached, particularly during the final two weeks. In contrast, NORM R and TCARI were generally higher in PWD-consistent observations, with the largest group differences also occurring near first visible discoloration.

Overall, the VI trajectories indicate that pooled group-level spectral differences emerged from approximately seven weeks before first visible crown discoloration, while the separation between healthy and PWD-consistent observations became more pronounced during the later pre-visual period, especially during the final two weeks.

The temporal progression was further supported by the supplementary effect-size analysis. The mean absolute rank-biserial effect size across the ten selected VIs was 0.067 at −8 weeks, followed by 0.221 and 0.202 at −7 and −6 weeks, respectively. It reached 0.343, 0.454, and 0.444 at −5, −4, and −3 weeks, respectively, before increasing markedly to 0.627 at −2 weeks and 0.797 at −1 week. Although the week-to-week progression was not strictly monotonic, the overall effect-size trajectory showed an early but relatively modest group-level separation followed by substantially stronger spectral divergence as first visible crown discoloration approached. Complete feature-specific estimates and tree-cluster bootstrap 95% confidence intervals are provided in Supplementary Table S1.

### 2.4. Feature-Specific Detection Rates Before First Visible Discoloration

**Figure 4:**
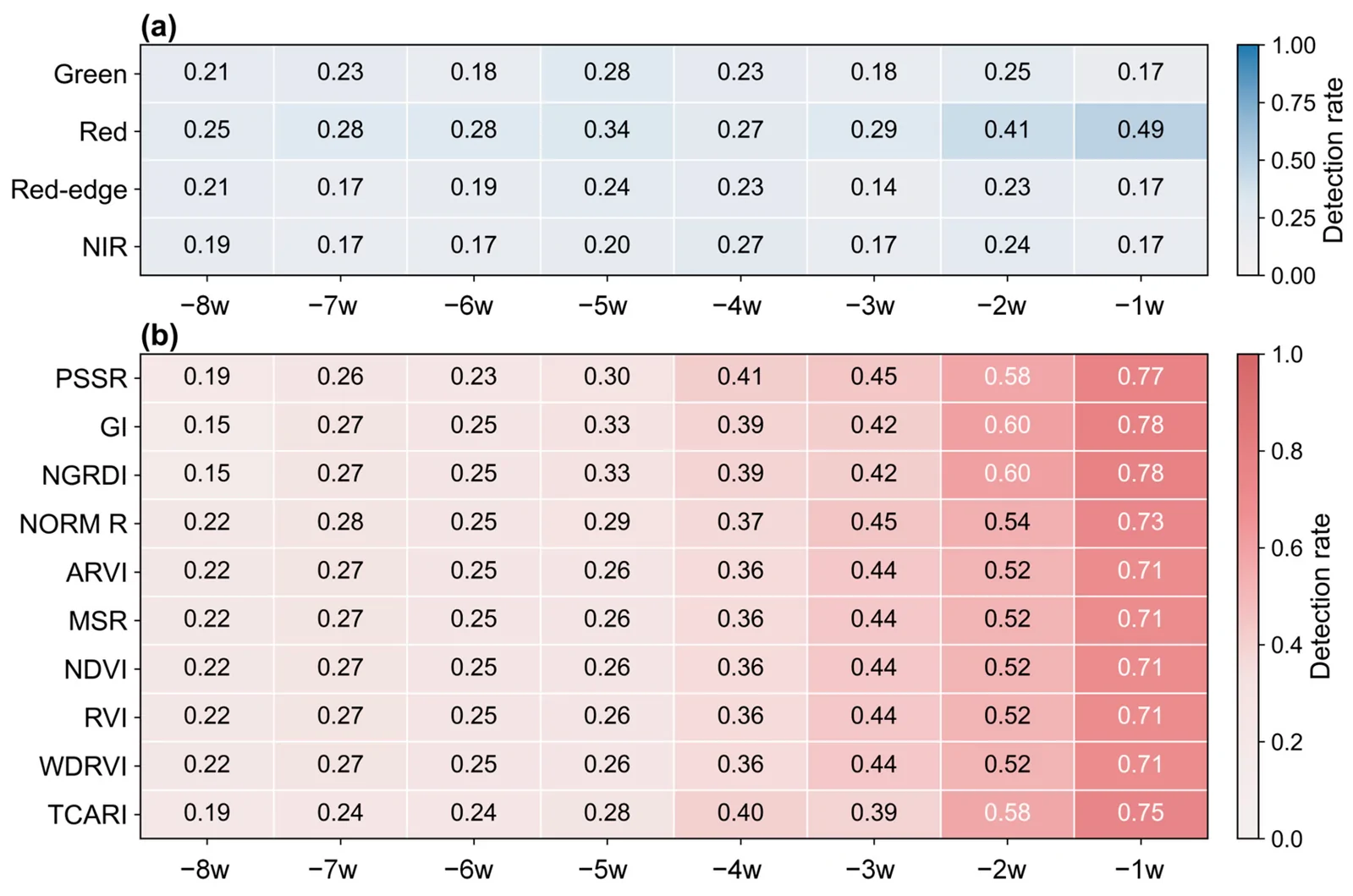
DR heatmaps of band reflectance and VIs. (**a**) Heatmap of band reflectance; (**b**) heatmap of the selected VIs.

Feature-specific DRs calculated using the 5th–95th percentile healthy reference intervals differed markedly between band reflectance and VIs (Figure 4). The DRs of the four individual bands fluctuated across relative times and did not increase consistently as first visible discoloration approached. Band DRs were relatively high eight weeks before first visible discoloration, declined at four and three weeks before discoloration, and increased partially during the final week. At one week before first visible discoloration, the band DRs ranged from 0.26 to 0.35. Complete feature-specific DR estimates, healthy baseline values, and Wilson 95% confidence intervals across all relative weeks are provided in Supplementary Table S2.

In contrast, the selected VIs exhibited a more structured temporal progression of spectral departure. During the early pre-visual response period (−7 to −6 weeks), the selected VIs first showed consistent group-level divergence from healthy observations, while VI-based DR remained relatively low, with mean values of 0.21 and 0.15, respectively. During the progressive spectral-divergence period (−5 to −3 weeks), group separation persisted and became more established, although the DR trajectory was not strictly monotonic; mean VI-based DR was 0.22, 0.28, and 0.23 at −5, −4, and −3 weeks, respectively. The clearest transition occurred during the advanced pre-visual deterioration period (−2 to −1 weeks, approaching week 0), when mean VI-based DR increased sharply to 0.52 and 0.68. Thus, the temporal sequence progressed from an early group-level spectral response, through sustained spectral divergence, to a substantially more widespread departure from the healthy spectral range immediately before first visible crown discoloration.

### 2.5. Overall Detection-Rate Trends of Bands and VIs

**Figure 5:**
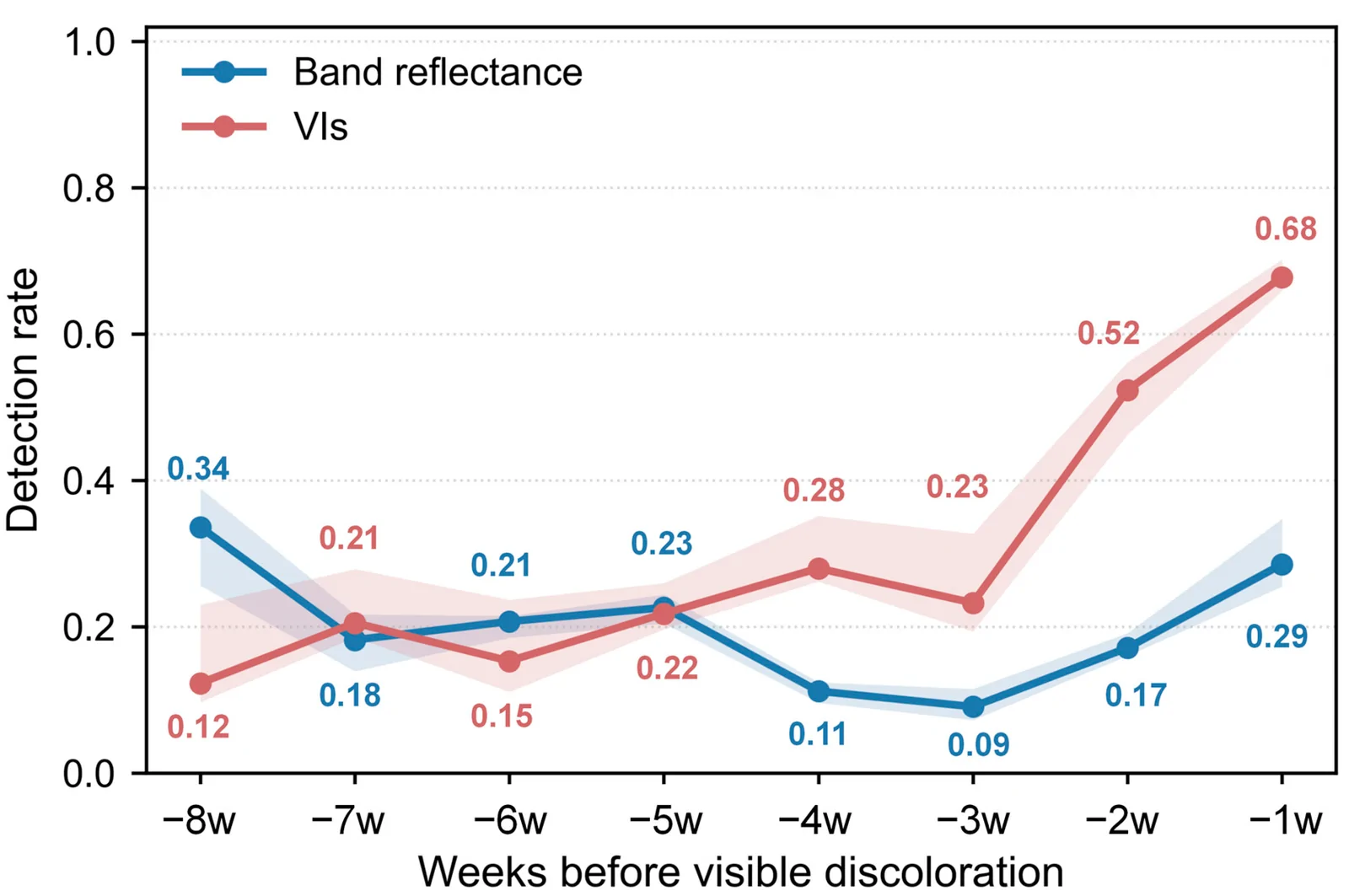
Overall trends in spectral separability of band reflectance and VIs based on DR. Lines indicate the mean DR of each feature group across retrospective weeks; numbers beside the points indicate mean DR values; shaded areas indicate the minimum-to-maximum DR range among features within each group.

The descriptive mean DR of the four bands fluctuated substantially across the pre-visual period (Figure 5), with values of 0.34, 0.18, 0.21, 0.23, 0.11, 0.09, 0.17, and 0.29 from −8 to −1 weeks, respectively. In contrast, the corresponding mean DR values of the selected VIs were 0.12, 0.21, 0.15, 0.22, 0.28, 0.23, 0.52, and 0.68.

Considered together with the week-specific significance and supplementary effect-size results, these patterns indicate a progressive temporal strengthening of the pre-visual spectral response. Group-level VI differences emerged approximately seven weeks before first visible crown discoloration, whereas the magnitude of spectral separation and the proportion of PWD-consistent observations outside the healthy reference range increased more strongly as visible discoloration approached, particularly during the final two weeks. These temporal patterns describe the progressive development of spectral departure rather than statistically estimated change points or directly demonstrated physiological stages.

## 3. Discussion

### 3.1. Methodological Value of Onset-Aligned Analysis

A central challenge in the pre-visual monitoring of PWD is that individual trees do not reach visible crown discoloration at the same time. Previous multi-temporal studies have commonly organized observations according to acquisition date, predefined disease stage, or sample class. Although these approaches are suitable for comparing spectral features or classification performance at particular dates, calendar-based grouping may combine trees located at different positions relative to their first visible crown change [15,17]. Consequently, subtle pre-visual responses may be diluted when trees with different symptom-onset times are analyzed together.

In this study, the first visible crown discoloration of each subsequently discolored tree was used as an individual temporal anchor. UAV observations acquired during the preceding eight monitoring weeks were reorganized according to their relative time before this event. This onset-aligned framework does not determine the actual time of infection or the complete physiological course of PWD; rather, it standardizes observations according to a clearly identifiable phenotypic endpoint. It therefore permits a more direct examination of when population-level spectral differences first become detectable and how those differences change as visible discoloration approaches.

The value of this approach lies in distinguishing a disease-relative warning window from a calendar-based monitoring period. A favorable calendar date may vary among sites, years, and individual trees because of differences in infection timing, weather, phenology, and disease development. In contrast, alignment to first visible discoloration addresses the practical question of how much lead time spectral monitoring may provide before an obvious crown response is observed. The present results indicate that this lead time cannot be represented by a single threshold: statistically detectable group differences appeared considerably earlier than the marked increase in the proportion of individual trees outside the healthy spectral range.

### 3.2. Stage-Dependent Spectral Responses and the Sensitivity of VIs

The spectral response preceding visible crown discoloration was feature dependent. Among the individual bands, red reflectance showed the most recurrent differences between healthy and PWD-consistent observations, whereas green, red-edge, and NIR reflectance varied more irregularly across relative weeks. Increased red reflectance is consistent with a reduction in red-light absorption as canopy pigment condition deteriorates. However, because chlorophyll concentration and other physiological traits were not measured directly, this interpretation should be considered a plausible spectral explanation rather than direct evidence of pigment loss. Altered stomatal regulation and canopy water relations may also contribute to pre-visual spectral changes during PWD development. However, direct attribution to reduced transpiration or evapotranspiration would require thermal or physiological measurements that were not available in the present experiment. Future work should further examine the physiological processes underlying the observed spectral sequence. In particular, integrating UAV multispectral and thermal observations with concurrent measurements of secondary-defense metabolites, stomatal conductance, leaf or canopy water status, transpiration-related traits, pigment condition, and other physiological variables would help determine how progressive changes in defense, hydraulic function, and canopy water exchange are related to the pre-visual spectral response. Thermal or evapotranspiration-related indicators should also be evaluated when the required thermal and meteorological measurements are available.

In comparison with individual bands, the selected VIs exhibited more persistent group-level differences. ARVI, GI, MSR, NDVI, NGRDI, PSSR, RVI, and WDRVI were generally lower in PWD-consistent symptomatic trees, whereas NORM R and TCARI were generally higher. These contrasting directions indicate that the pre-visual response was not restricted to a single wavelength but involved changes in the relative relationships among visible, red-edge, and NIR reflectance. Ratio- and normalized-difference indices can partly suppress common variations in illumination and overall reflectance magnitude while emphasizing relative changes among spectral bands, which may explain their greater temporal consistency compared with raw band reflectance. Similar advantages of VIs over individual reflectance bands have also been reported in UAV-based studies of forest stress [19,20,21].

Previous PWD studies using multi-temporal UAV imagery have similarly shown that spectral responses and detection performance vary with acquisition date and disease stage [15,16,17,18]. However, most reported monitoring windows were defined according to calendar acquisition dates or broad symptom stages rather than the tree-specific interval preceding first visible discoloration. For example, Yu et al. [15] reported hyperspectral changes in June before evident field discoloration was observed in August, whereas Wu et al. [16] identified late July as a favorable early monitoring period for the green-attack stage of PWD. In a related forest-health system, Huo et al. [22] reported that multispectral detectability of European spruce bark beetle attack increased from approximately 15% after five weeks of infestation to approximately 90% after ten weeks. These temporal estimates are not directly interchangeable because the studies differ in temporal anchor, host–stress system, sensor, and definition of detectability. Nevertheless, they collectively indicate that remotely sensed forest-stress signals can develop progressively before conspicuous symptoms or advanced crown deterioration. The present onset-aligned analysis extends this temporal perspective by expressing spectral responses relative to each tree’s first visible crown discoloration, with pooled group-level VI divergence emerging approximately 6–7 weeks before visible discoloration and substantially stronger and more widespread spectral departure occurring during the final two pre-visual weeks.

Within this onset-aligned sequence, the onset-aligned population-level spectral pattern showed an overall temporal progression. At approximately −7 to −6 weeks, consistent group-level VI divergence first emerged while VI-based DR remained relatively low, indicating the earliest detectable spectral response in the present dataset. This early divergence is consistent with subtle spectral consequences of developing physiological stress before obvious crown symptoms. As visible symptom onset approached, the VI response subsequently amplified, with the most pronounced change occurring at −2 to −1 weeks. Mean VI-based DR increased sharply to 0.52 and 0.68, respectively, indicating that spectral abnormalities had become substantially more widespread among subsequently discolored trees. This final pre-visual period is therefore more consistent with accelerated physiological deterioration immediately preceding visible symptom development, whereas week 0 marks the onset of visible crown discoloration. Accordingly, the early-detection significance of the present results lies in the emergence of VI divergence approximately 6–7 weeks before visible symptoms, while the high DR during the final two weeks reflects the subsequent intensification of the pre-visual spectral response rather than its earliest detectable stage. Because chlorophyll concentration, leaf water status, and other physiological traits were not measured concurrently, the proposed physiological progression remains an interpretation of the observed spectral sequence rather than a directly measured physiological trajectory.

The isolated −8-week band response may partly reflect the greater sensitivity of raw reflectance to acquisition conditions, site background, sample composition, illumination, and crown structure. Its biological significance as an early response should therefore be interpreted cautiously. The later increase in VI-based DR occurred across multiple indices and was most pronounced during the final two pre-visual weeks, providing more coherent evidence of progressive spectral departure.

### 3.3. Progressive Strengthening of the Pre-Visual Warning Window and Its Practical Implications

Considered together, the significance, effect-size, and DR results indicate a progressive rather than sharply segmented pre-visual spectral response. In the pooled analysis, consistent group-level differences among the selected VIs emerged at approximately −7 to −6 weeks, while the magnitude of separation remained relatively modest. Mean absolute rank-biserial effect size was 0.221 and 0.202 at −7 and −6 weeks, respectively, and mean VI-based DR remained relatively low at 0.21 and 0.15. The approximately seven-week result should therefore be interpreted as an early pooled group-level indication of spectral divergence rather than as a universal individual-tree detection boundary. For practical interpretation, this progression can be described using three descriptive operational periods: an early pre-visual response period at approximately −7 to −6 weeks, a progressive spectral-divergence period from −5 to −3 weeks, and an advanced pre-visual deterioration period at −2 to −1 weeks.

During the progressive spectral-divergence period (−5 to −3 weeks), the differences between healthy and PWD-consistent observations persisted, indicating that the spectral response had become more established. Mean VI-based DR was 0.22, 0.28, and 0.23 at −5, −4, and −3 weeks, respectively. Although these values did not increase monotonically from week to week, the persistence of group-level VI separation across successive observations supports the interpretation of a developing rather than transient spectral response.

The strongest change occurred during the advanced pre-visual deterioration period (−2 to −1 weeks, approaching week 0). Mean VI-based DR increased sharply to 0.52 and 0.68, indicating that spectral departure from the healthy reference range had become substantially more widespread among subsequently discolored trees. This final period should not be regarded as the earliest detectable stage of disease-associated stress; rather, it represents the period in which the pre-visual spectral response became strongest immediately before the first visible crown discoloration.

These temporal periods have different operational implications. The early response at approximately −7 to −6 weeks is most appropriately treated as a warning indication that may justify continued or intensified UAV surveillance rather than confident identification of individual affected trees. Persistent abnormalities during −5 to −3 weeks may strengthen evidence for repeated risk flagging when similar deviations recur across successive acquisitions. The final −2 to −1 week period provides a stronger basis for prioritizing particular crowns or areas for field inspection and confirmatory sampling because a substantially larger proportion of subsequently discolored trees falls outside the healthy spectral reference range.

These three periods are descriptive operational intervals derived from the observed temporal pattern, rather than statistically estimated change points or directly demonstrated physiological stages. Accordingly, the warning window should not be represented as a single universal detection date or as a set of sharply separated biological stages. Instead, the results support a progressive monitoring framework in which subtle group-level spectral divergence emerges first and becomes increasingly widespread as visible crown discoloration approaches. DR should therefore be interpreted as a descriptive measure of departure from the healthy spectral reference range rather than as classification accuracy, diagnostic sensitivity, or proof of PWD infection.

### 3.4. Limitations and Future Perspectives

Several limitations should be considered when interpreting these results. First, sample status was primarily determined through sequential image interpretation and the subsequent development of visible crown discoloration, wilting, or mortality. Healthy reference trees were followed into the subsequent year and were included only when no visible crown abnormality developed, which reduced the risk of including trees with delayed discoloration. Nevertheless, pathogen infection was not confirmed individually for all analyzed trees. The spectral patterns reported here should therefore be interpreted as responses associated with trees subsequently exhibiting PWD-consistent crown symptoms rather than as direct pathogen-diagnostic signatures. Other biotic or abiotic stressors capable of altering crown pigment condition, water relations, or canopy structure may produce partially overlapping spectral responses. Future studies integrating nematode detection, field symptom assessment, and physiological measurements would strengthen the biological interpretation of pre-visual spectral changes.

Second, first visible discoloration was determined from sequential UAV images acquired at an approximately weekly frequency. The actual onset of visible crown change may have occurred between two acquisitions, producing temporal uncertainty of up to approximately one monitoring interval. The missed acquisition in mid-July further reduced temporal continuity for some trees and contributed to differences in usable sample numbers among relative weeks. Accordingly, the approximate timing assigned to the early response (−7 to −6 weeks), progressive divergence (−5 to −3 weeks), and strongest pre-visual deterioration (−2 to −1 weeks) should be interpreted as descriptive monitoring periods rather than exact temporal boundaries. The transitions among these periods may have occurred between consecutive UAV surveys. Higher-frequency UAV acquisition combined with field observations would allow the timing and continuity of these spectral changes to be resolved more precisely.

Third, the composition and number of usable observations varied among relative weeks because of image availability and crown-level quality control. In addition, healthy reference observations were assembled from the acquisition-date strata represented at each relative week, such that the same healthy tree could contribute more than one tree-time observation within a relative-week dataset when it served as a reference in multiple acquisition-date strata. Tree identities could also recur across relative weeks. Consequently, although the primary analyses were conducted separately at each relative week, the nominal independence assumption of the week-specific Mann–Whitney U tests may be partially violated, particularly for the healthy reference observations. The resulting significance tests should therefore be interpreted as exploratory population-level evidence of onset-aligned group differences rather than as formal longitudinal inference at the individual-tree level, and the associated *p* values and FDR-adjusted significance levels should be interpreted with this limitation in mind. Tree identity was retained as the clustering unit in the supplementary bootstrap analyses, but these robustness analyses do not eliminate the dependence present in the primary week-specific comparisons or replace a formal repeated-measures model. Future studies using more complete longitudinal sequences and mixed-effects or related repeated-measures models would provide stronger within-tree evidence of temporal change.

Finally, the study was conducted at two *Pinus massoniana* forest sites during a single monitoring season. Stand structure, terrain, phenology, meteorological conditions, sensor configuration, and disease pressure may alter both the spectral response and the timing of visible discoloration. In addition, the magnitude and timing of early VI divergence were not identical between the two study sites, indicating that the earliest spectral response may depend on site-specific environmental conditions, stand characteristics, and differences in disease progression among trees. Therefore, the approximately seven-week signal identified in the pooled analysis should be regarded as an exploratory group-level indication rather than a universal temporal boundary. Although site- and acquisition-date-matched comparisons reduced variation associated with shared acquisition and environmental conditions, meteorological variables were not modeled explicitly; residual environmentally induced variation therefore cannot be completely excluded. Future work should test the onset-aligned framework across additional regions, years, pine species, and sensor systems. Integration of UAV time series with pathogen confirmation, thermal observations, and concurrent measurements of pigment condition, water status, stomatal regulation, and other physiological traits would be particularly valuable for examining how the three operational spectral periods relate to changes in tree physiological condition.

## 4. Materials and Methods

### 4.1. Study Area

**Figure 6:**
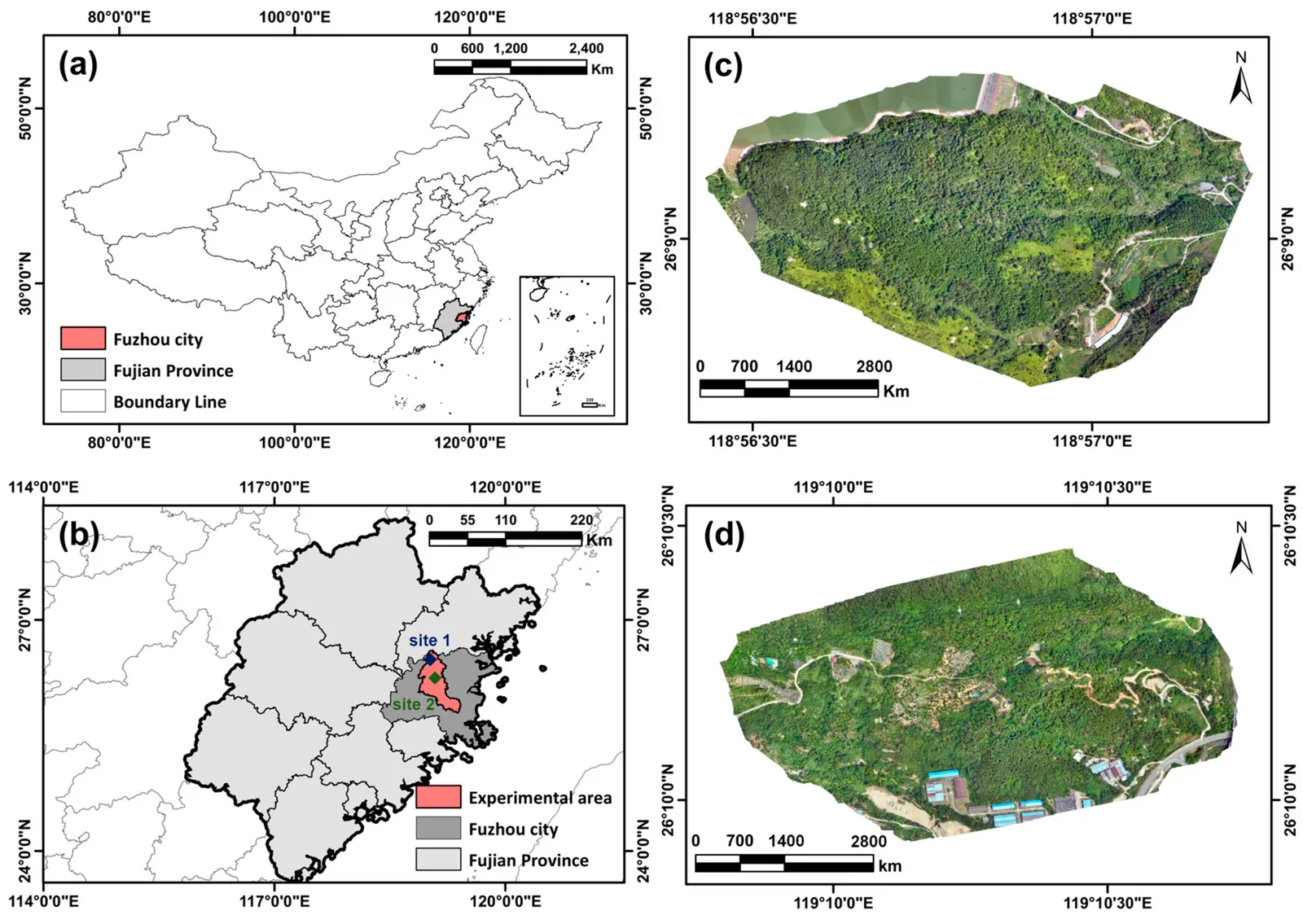
Study area. (**a**) Location of Fujian Province, with Fuzhou City highlighted in red; (**b**) location of the study area, with the experimental area highlighted in red; (**c**) true-color UAV orthomosaic of Site 1; and (**d**) true-color UAV orthomosaic of Site 2.

The study sites were located in Minhou County, Fuzhou City, Fujian Province, southeastern China (Figure 6). Two sites were established: Site 1 (26°8′49″ N, 118°58′12″ E, Figure 6c) and Site 2 (26°9′55″ N, 119°8′8″ E, Figure 6d). The region is characterized by hilly terrain typical of southern China and has a subtropical maritime monsoon climate. The mean annual temperature is approximately 19.5 °C, and the mean annual precipitation is approximately 1674 mm. The forest stands are mixed forests dominated by *Pinus massoniana*.

### 4.2. UAV-Based RS Data Acquisition and Preprocessing

UAV multispectral imagery was acquired using a DJI Mavic 3 Multispectral UAV (DJI, Shenzhen, China), equipped with one 20-megapixel RGB camera and four 5-megapixel multispectral cameras. The wavelength ranges of the multispectral bands are listed in Table 2. Flights were conducted between 10:00 and 14:00, preferentially around midday, under clear and rain-free conditions without obvious cloud interference. A flight altitude of 200 m and a flight speed of 9.7 m/s were maintained, with 80% forward overlap and 70% side overlap, resulting in a ground sampling distance of approximately 9.22 cm/pixel. Scheduling acquisitions near midday and under comparable weather conditions helped reduce variation in solar elevation and illumination among monitoring dates. During the June–August 2025 monitoring period, air temperature was generally 30–33 °C, and no apparent drought occurred at either study site. The region has a warm and humid subtropical monsoon climate, with relatively high precipitation and humidity during summer; therefore, the monitoring period did not coincide with an evident drought event likely to induce substantial drought-related changes in canopy reflectance.

UAV monitoring was conducted approximately once per week from early June to August 2025, although the exact acquisition dates differed slightly between the two study sites. At Site 1, imagery was acquired on 5, 11, 20, and 26 June; 3, 18, and 24 July; and 1, 7, and 12 August 2025. At Site 2, imagery was acquired on 3, 10, 17, and 25 June; 3, 19, 25, and 31 July; and 7 and 12 August 2025. The planned acquisitions between 7 and 13 July were not completed because weather conditions were unsuitable for UAV operation. Additional imagery was acquired on 5 September at Site 1 and 1 September at Site 2 to confirm the final crown-discoloration or mortality status of individual trees; these September observations were not included in the pre-visual spectral time-series analysis.

Images from each flight mission were processed using DJI Terra (version 4.5, DJI, Shenzhen, China). Radiometric calibration was conducted before and after each flight using a calibrated reflectance reference panel, and the acquired multispectral imagery was converted to surface reflectance before subsequent spectral analysis. Consistent acquisition settings and similar flight times were maintained across monitoring dates to reduce variation associated with illumination geometry. All images were processed using the same photogrammetric workflow, including image alignment, orthomosaic generation, and geometric correction. The resulting RGB and multispectral orthomosaics were co-registered to a common spatial reference to ensure consistent correspondence of individual trees and crown regions across acquisition dates. The green, red, red-edge (RE), and near-infrared (NIR) orthomosaics were subsequently stacked in ENVI 5.3 (Harris Corporation, Melbourne, FL, USA), and the resulting four-band composite images were used for crown-level spectral extraction.

### 4.3. Construction of the Onset-Aligned Dataset

Individual-tree crowns were manually delineated as regions of interest (ROIs) using ENVI 5.3. Trees that subsequently exhibited pronounced crown discoloration, wilting, or mortality consistent with the field expression of PWD were operationally designated as PWD-consistent symptomatic trees for the onset-aligned analysis. Because individual pathogen confirmation was not available for every tree, this designation refers to the observed crown trajectory rather than to a pathogen-confirmed diagnosis. Each sample corresponded to an independent crown ROI. Crown identity and ROI correspondence were checked across sequential georeferenced orthomosaics using crown position, shape, and surrounding canopy features as spatial references. For each monitoring date, the ROI was visually checked against the corresponding crown position, and minor positional discrepancies associated with crown displacement or image co-registration were corrected where necessary. Observations for which crown correspondence could not be established reliably because of displacement, image misregistration, or insufficient crown visibility were excluded from subsequent analysis. Continuous interpretation of the time-series imagery was then used to identify the first calendar week in which visible crown discoloration was observed for each PWD-consistent symptomatic tree.

The calendar week of first visible crown discoloration was defined as week 0. First visible crown discoloration was operationally defined as the first monitoring week in which a previously green crown exhibited visually recognizable discoloration in the sequential UAV RGB imagery. Observations obtained during the preceding eight monitored calendar weeks were retrospectively assigned to the corresponding relative weeks from −8 to −1. These relative-week labels represent weekly monitoring periods relative to the first visible crown discoloration rather than exact seven-day intervals. A tree observation was included in a relative-week dataset when the crown could be reliably matched on the corresponding image and complete spectral information was available. Crown-level quality control was conducted for every retained tree-time observation. Observations affected by obvious image artefacts, severe shading, incomplete crown coverage, background contamination, unreliable temporal crown correspondence, or invalid spectral values were excluded from subsequent analysis.

Healthy reference trees were selected from individuals that remained free of visible crown discoloration, wilting, or mortality throughout the 2025 monitoring campaign. Their status was further monitored through June 2026 using repeated UAV imagery and field inspections, and only trees that remained free of visible crown abnormalities during this follow-up were retained as healthy references. Within each study site, healthy ROIs were selected sequentially according to their pre-established ROI order. The ROI order was established during crown delineation and was unrelated to band reflectance, VI values, or subsequent statistical results.

For each relative week, the healthy reference set was constructed from the same study-site and acquisition-date strata represented by the corresponding PWD-consistent observations. This design reduced variation associated with acquisition conditions, seasonal phenology, site background, and image-processing batches while preserving the original onset-aligned comparison framework. Because no valid imagery was available during calendar week 28 and not all crown observations satisfied the quality-control criteria at every retrospective time, the number of usable observations varied among relative weeks, and the numbers of healthy and PWD-consistent observations were not necessarily identical at every relative week. The final week-specific sample sizes used in the onset-aligned analysis are summarized in Table 3.

### 4.4. Spectral Feature Extraction and VI Selection

For each individual-tree ROI and acquisition date, valid crown pixels were defined as pixels within the accepted crown ROI for which valid reflectance values were available in all four multispectral bands. No additional pixel-level shadow threshold or systematic crown-edge erosion was applied. Instead, observations affected by severe shading, obvious background contamination, incomplete crown coverage, image artefacts, or unreliable temporal crown correspondence were excluded during crown-level quality control. The mean reflectance of the retained crown pixels was then calculated separately for the green, red, RE, and NIR bands. These four crown-level mean reflectance values were used as the spectral vector for each tree-time observation, and the candidate VIs were subsequently calculated from these mean band-reflectance values. This approach was selected because the analytical unit of the present study was the individual crown, and it provided one consistent crown-level spectral vector for each tree-time observation while reducing the influence of fine-scale pixel variability and minor spatial inconsistencies among repeated acquisitions. This crown-level aggregation approach has been widely used in comparable individual-tree UAV forest-health studies [4,8,22].

To reduce the number of candidate indices and retain representative VIs showing relatively strong class-associated responses, sample status was encoded as a binary variable, and Spearman’s rank correlation coefficient was calculated between each candidate VI and sample status. The VIs were ranked according to the absolute correlation coefficient (|ρ|). The *p* values associated with the 41 correlation tests were adjusted using the Benjamini–Hochberg false discovery rate procedure, and adjusted *p* values below 0.05 were considered statistically significant. The ten highest-ranked VIs were retained for subsequent time-series, significance, and DR analyses. This procedure was used as an exploratory feature-ranking step rather than as a classification model or an independent assessment of predictive performance. Because the selected VIs were ranked using their association with sample status in the same dataset, subsequent temporal patterns of the Top-10 VIs were interpreted as exploratory and may preferentially reflect indices showing stronger or more temporally consistent responses within the present dataset. The definitions, formulas, and references of the candidate VIs are provided in Table 4.

To evaluate the stability of this exploratory ranking, the feature-ranking procedure was additionally repeated in 100 tree-level bootstrap resamples stratified by sample status and study site. All observations belonging to a resampled tree were retained together within the same bootstrap unit.

### 4.5. Detection Rate (DR) Calculation

A healthy-reference-based detection rate (DR) was calculated following the detectability framework described by Huo et al. [22]. For each relative week and each spectral feature, the 5th and 95th percentiles of the healthy observations in that relative-week dataset were used as the lower and upper limits of the healthy reference interval, respectively. Each PWD-consistent observation was then compared with the corresponding week-specific healthy reference interval and was considered spectrally abnormal when its feature value was lower than the 5th percentile or higher than the 95th percentile of the healthy distribution. For each feature and each time before first visible discoloration, DR was calculated as

*DR* = *n*_outside_/*n*_PWD_

where *n_outside_* is the number of PWD-consistent observations outside the corresponding week-specific healthy reference interval and *n_PWD_* is the total number of PWD-consistent observations at that relative time. DR therefore represents the proportion of subsequently discolored trees exhibiting spectral values outside the healthy reference range and was treated as a descriptive measure of spectral departure rather than as classification accuracy, diagnostic sensitivity, or proof of PWD infection.

To characterize the out-of-range rate expected among healthy observations without evaluating an observation against a reference interval to which it had itself contributed, an empirical healthy baseline was additionally estimated using a leave-one-out procedure. For each healthy observation, the 5th–95th percentile reference interval was recalculated from the remaining healthy observations for the same relative week and spectral feature, and the held-out observation was then classified as within or outside that interval. Wilson 95% confidence intervals were additionally calculated for feature-specific PWD DR estimates. Mean DR values across the four single bands and across the ten selected VIs were calculated only as descriptive summaries of the corresponding feature groups and were not interpreted as independent evidence or diagnostic performance.

### 4.6. Statistical Analysis and Visualization

For each spectral feature and each relative week before first visible crown discoloration, differences between healthy and PWD-consistent observations were evaluated using two-sided Mann–Whitney U tests. *p* values obtained across the eight relative weeks were adjusted using the Benjamini–Hochberg false discovery rate procedure. Adjusted *p* values below 0.05 were considered statistically significant. As a supplementary robustness analysis, signed rank-biserial correlation (*r*_rb) was calculated to quantify the magnitude and direction of group separation for the selected VIs. Negative *r*_rb values indicate lower feature values in PWD-consistent observations than in healthy observations, whereas positive values indicate higher values. Ninety-five percent confidence intervals for *r*_rb were estimated using 500 tree-cluster bootstrap resamples stratified by sample status and study site, with tree identity as the resampling unit. These effect-size analyses were used as supplementary robustness assessments, while Welch’s *t*-tests remained the primary week-specific comparisons.

For PWD-consistent observations, each tree contributed at most one observation within a given relative-week dataset. Healthy reference observations were assembled from the acquisition-date strata represented at each relative week; consequently, the same healthy tree could contribute more than one tree-time observation within a relative-week dataset when it served as a reference in multiple acquisition-date strata. Tree identities could also recur across relative weeks. Consequently, although the primary analyses were conducted separately at each relative week, the nominal independence assumption of the week-specific Mann–Whitney U tests may be partially violated, particularly for the healthy reference observations. The resulting significance tests were therefore interpreted as exploratory population-level evidence of onset-aligned group differences rather than as formal longitudinal inference at the individual-tree level, and the associated *p* values and FDR-adjusted significance levels should be interpreted with this limitation in mind. Tree identity was retained as the clustering unit in the supplementary bootstrap analyses to account for repeated contributions within those robustness assessments; however, these supplementary analyses do not eliminate the dependence present in the primary week-specific comparisons.

**Figure 7:**
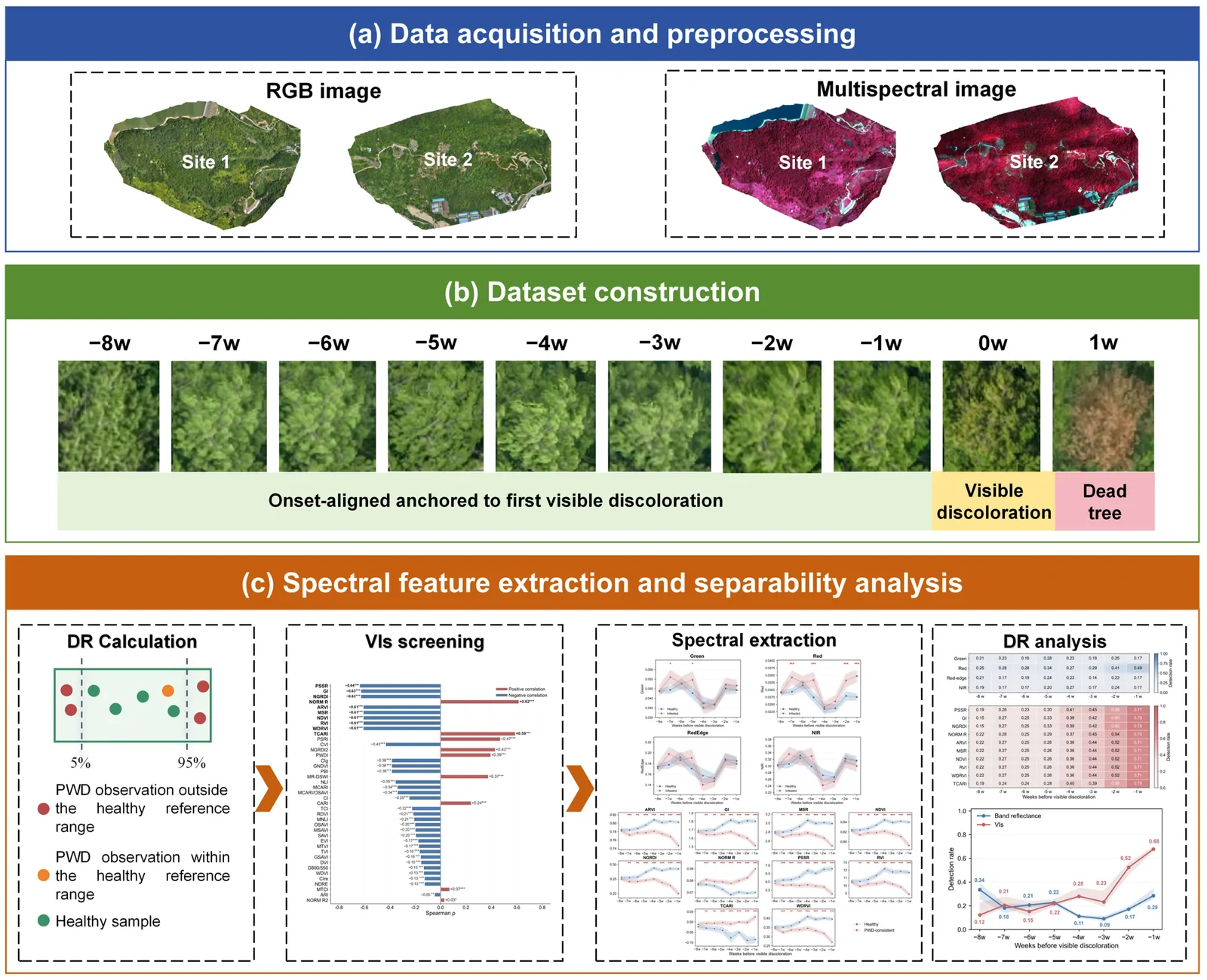
Technical workflow. (**a**) UAV image acquisition and preprocessing; (**b**) construction of the onset-aligned dataset; and (**c**) spectral feature extraction and analysis. Asterisks indicate BH–FDR-adjusted significance levels: *, adjusted *p* < 0.05; **, adjusted *p* < 0.01; and ***, adjusted *p* < 0.001. Shaded areas in the temporal plots represent 95% confidence intervals, whereas those in the overall DR-trend plot represent the minimum-to-maximum range among features within each group.

Spearman correlation analysis was used for exploratory VI ranking, and the *p* values from the 41 candidate-VI correlations were separately corrected using the Benjamini–Hochberg procedure. Time-series plots of band reflectance and VIs show the arithmetic mean and corresponding 95% confidence interval for each group. DR heatmaps show the proportion of PWD-consistent observations outside the corresponding week-specific healthy reference range. In the feature-group summary plot, lines represent the mean DR across the features within each group, whereas shaded areas represent the minimum-to-maximum DR range among those features and should not be interpreted as statistical confidence intervals. Data processing, statistical analysis, and visualization were performed in Python 3.12 using pandas 2.2.2, NumPy 1.26.4, SciPy 1.13.1, and Matplotlib 3.9.2. The overall analytical workflow is shown in Figure 7.

## 5. Conclusions

This study used the first visible crown discoloration of each subsequently discolored tree as an individual temporal anchor to characterize pre-visual spectral responses associated with PWD-consistent crown symptom development using weekly UAV multispectral observations. Individual-band reflectance showed considerable temporal fluctuation, whereas the selected VIs provided more consistent separation between healthy and PWD-consistent samples. The onset-aligned analysis revealed a progressive population-level pre-visual spectral pattern: consistent group-level VI divergence emerged at approximately −7 to −6 weeks, persisted through a period of progressive spectral divergence from −5 to −3 weeks, and became substantially more widespread during the final −2 to −1 weeks, when mean VI-based DR increased to 0.52 and 0.68, respectively. The early response should be interpreted as a pooled warning indication rather than reliable individual-tree detection, whereas the final two pre-visual weeks provide a stronger basis for prioritizing crowns for field inspection and confirmatory sampling. These temporal periods describe the observed progression of spectral departure rather than statistically discrete physiological stages. The onset-aligned framework therefore provides a practical basis for repeated UAV surveillance by distinguishing early spectral indication from the later strengthening of individual-tree abnormality. DR should be interpreted as a descriptive measure of spectral departure from the healthy reference range rather than as classification accuracy, diagnostic sensitivity, or direct disease diagnosis.

## Figures and Tables

**Table 1:** Stability of the selected VIs under 100 tree-level bootstrap resamples.

VI	Full-Data Rank	Top-10 Inclusion Frequency
PSSR	1	100%
GI	2	100%
NGRDI	3	100%
NORM R	4	100%
ARVI	5	100%
MSR	6	100%
NDVI	7	100%
RVI	8	100%
WDRVI	9	98%
TCARI	10	99%

**Table 2:** Spectral bands of the multispectral camera.

Band	Wavelength
Green	544–576 nm
Red	634–666 nm
RE	714–746 nm
NIR	834–886 nm

**Table 3:** Numbers of healthy and PWD-consistent tree observations included at each relative week before first visible crown discoloration.

Relative Week	Healthy Observations	PWD-Consistent Observations
−8 w	113	113
−7 w	129	129
−6 w	135	135
−5 w	127	127
−4 w	145	145
−3 w	165	165
−2 w	162	162
−1 w	141	141

Note: The same tree could contribute observations to more than one relative-week dataset.

**Table 4:** Candidate VIs used in this study.

Indices	Formula	Reference
ARI (Anthocyanin Reflectance Index)	1/GREEN − 1/RE	[23]
ARVI (Atmospherically resistant vegetation index)	1.17 × [(NIR − RED)/(NIR + RED)] − 0.18	[24]
CARI (Chlorophyll Absorption Ratio Index)	RE × SQRT(a × 650 + RED + b)/RED × SQRT (a^2^ + 1), a = (RE − GREEN)/170; b = GREEN − (a × 560)	[25]
CI (Chlorophyll Index)	(NIR − RE)/(NIR − RED)	[26]
CIg (Chlorophyll Index Green)	NIR/GREEN − 1	[27]
CIre (Chlorophyll Index Red-edge)	NIR/RE − 1	[28]
CVI (Chlorophyll Vegetation Index)	(NIR × RED)/(GREEN × GREEN)	[29]
D800/550 (Difference 800/550)	NIR − GREEN	[30]
DVI (Difference Vegetation Index)	NIR − RED	[31]
EVI (Enhanced Vegetation Index)	2.4 × (NIR − RED)/(NIR + RED + 1)	[32]
GI (Greenness Index)	GREEN/RED	[33]
GNDVI (Green NDVI)	(NIR − GREEN)/(NIR + GREEN)	[34]
GSAVI (Green Soil-Adjusted Vegetation Index)	1.5 × (NIR − GREEN)/(NIR + GREEN + 0.5)	[35]
MCARI (Modified Chlorophyll Absorption Ratio Index)	[(RE − RED) − 0.2 × (RE − GREEN)] × (RE/RED)	[36]
MCARI/OSAVI	MCARI/OSAVI	[36]
MNLI (Modified Non-Linear Vegetation Index)	1.5 ×(NIR^2^ − RED)/(NIR^2^ + RED + 0.5)	[37]
MR-DSWI (Multiple Ratio Disease–Water Stress Index)	(RE^2^ × RED)/(GREEN^2^ × RE)	[22]
MSAVI (Improved Soil Adjusted Vegetation Index)	0.5{2 × NIR + 1 − SQRT[(2 × NIR + 1)2 − 8 (NIR − RED)]}	[38]
MSR (Modified Simple Ratio)	(NIR/Red − 1)/SQRT(NIR/Red + 1)	[37]
MTCI (MERIS Terrestrial Chlorophyll Index)	(NIR − RE)/(RE − RED)	[39]
MTVI (Modified Triangular Vegetation Index)	1.2 × [1.2 × (NIR − GREEN) − 2.5 × (RED − GREEN)]	[40]
NDVI (Normalised Difference Vegetation Index)	(NIR − RED)/(NIR + RED)	[41]
NDRE (Normalized Difference Red-edge Index)	(NIR − RE)/(NIR + RE)	[42]
NGRDI (Normalized Green Red Difference Index)	(GREEN − RED)/(GREEN + RED)	[41]
NGRDI2	(GREEN − RE)/(GREEN + RE)	[41]
NLI (Non-Linear Vegetation Index)	(NIR^2^ − RED)/(NIR^2^ + RED)	[37]
NORM R (Normalized Red Index)	RED/(GREEN + RED + NIR)	[43]
NORM R2 (Normalized Red-edge Index)	RE/(GREEN + RE + NIR)	[43]
OSAVI (Optimised Soil-Adjusted Vegetation Index)	1.16 × (NIR − RED)/(NIR + RED + 0.16)	[44]
PBI (Plant biochemical index)	NIR/GREEN	[45]
PSSR (Pigment Specific Simple Ratio)	RE/RED	[46]
RDVI (Renormalized Difference Vegetation Index)	(NIR − RED)/SQRT(NIR + RED)	[37]
RVI (Ratio Vegetation Index)	NIR/RED	[41]
PWDI (Pine wilt disease index)	−NGRDI × NDVI/GREEN	[47]
PSRI (Plant Senescence Reflectance Index)	(RE − RED)/GREEN	[48]
SAVI (Soil-adjusted Vegetation Index)	1.5 × (NIR − RED)/(NIR + RED + 0.5)	[49]
TCARI (Transformed Chlorophyll Absorption Reflectance Index)	3 × [(RE − RED) − 0.2 × (RE − GREEN) × (RE/RED)]	[50]
TCI (Triangular Chlorophyll Index)	1.2 × (RE − GREEN) − 1.5 × (RED − GREEN) × SQRT (RE/RED)	[51]
TVI (Triangular Vegetation Index)	60 × (NIR − GREEN) − 100 × (RED − GREEN)	[52]
WDRVI (Wide Dynamic Range Vegetation Index)	(0.2 × NIR − RED)/(0.2 × NIR + RED)	[53]
WDVI (Weighted Difference Vegetation Index)	NIR − 0.5 × RED	[54]

## Data Availability

The raw data supporting the conclusions of this article will be made available by the authors on request.

## Supplementary Materials

The following supporting information can be downloaded at: https://www.mdpi.com/article/10.3390/plants15172720/s1, Table S1: Signed rank-biserial effect sizes and tree-cluster bootstrap 95% confidence intervals for the ten selected VIs across all eight pre-visual relative weeks; Table S2: PWD detection rates, 95% confidence intervals, and healthy baselines across pre-visual weeks.
